# Supplementary material for: Spontaneous neuronal oscillations in the human insula are hierarchically organized traveling waves
Source: eLife. 2022 May 26;11:e76702. doi: 10.7554/eLife.76702 (PMC9200407; doi:10.7554/eLife.76702)
Supplement: Supplementary file 1. — (a). Wave-strength (denoted as ρadj2) of traveling waves is positively correlated with Hilbert-transformed amplitude of signals. We estimated the correlation between the average amplitude of signals of the electrodes and the wave-strength of traveling waves for each oscillation cluster. We used a circular-shuffling procedure to test the significance of correlations where we circularly shuffle the wave-strength values with respect to the average amplitude and estimate the correlation on this shuffled data to build a distribution of surrogate correlation values against which the observed correlation was tested (p<0.05) (Materials and methods). We found that for ~71% of clusters (20 out of the 28 clusters), there was a statistically significant correlation between amplitude and wave-strength. All, except one, clusters out of these 20 clusters, showed a statistically significant positive amplitude- ρadj2 correlation. Cluster frequency for an oscillation cluster was defined to be the average of the oscillation frequencies across all electrodes for the given oscillation cluster. (b). Wave-strength (denoted as ρadj2) of high (beta) frequency traveling waves is uncorrelated with the wave-strength (ρadj2 (low)- ρadj2 (high)), phase (phase (low)- ρadj2 (high)), and power (power (low)- ρadj2 (high)) of low (theta) frequency traveling waves. For testing the statistical significance of the observed correlations, values from one time-series were circularly shuffled with respect to the other so that the instantaneous correlation between the two time-series is destroyed, and correlation analysis was repeated on this shuffled data to build a distribution of surrogate correlation values against which the observed correlation was tested (p<0.05) (Materials and methods). [file elife-76702-supp1.docx]

**Supplementary File for**

**“Spontaneous Neuronal Oscillations in the Human Insula are Hierarchically Organized Traveling Waves”**

Anup Das^1*^, John Myers^2*^, Raissa Mathura^2^, Ben Shofty^2^, Brian Metzger^2^, Kelly Bijanki^2^, Chengyuan Wu^3^, Joshua Jacobs^1**^, Sameer A. Sheth^2**^

^1^Department of Biomedical Engineering, Columbia University, New York, NY 10027

^2^Department of Neurosurgery, Baylor College of Medicine, Houston, TX 77030

^3^Department of Neurosurgery, Thomas Jefferson University, Philadelphia, PA 19107

*These authors contributed equally to this work

**Joint senior authors

Correspondence:

Anup Das ([ad3772@columbia.edu](mailto:ad3772@columbia.edu)), Joshua Jacobs ([joshua.jacobs@columbia.edu](mailto:joshua.jacobs@columbia.edu)),

Department of Biomedical Engineering,

Columbia University,

New York, NY 10027

John Myers ([John.Myers@bcm.edu](mailto:John.Myers@bcm.edu)), Sameer A. Sheth ([Sameer.Sheth@bcm.edu](mailto:Sameer.Sheth@bcm.edu)),

Department of Neurosurgery,

Baylor College of Medicine,

Houston, TX 77030

**Supplementary tables**

**Supplementary File 1A.** **Wave-strength (denoted as**$\rho_{adj}^{2}$**) of traveling waves is positively correlated with Hilbert-transformed amplitude of signals.** We estimated the correlation between the average amplitude of signals of the electrodes and the wave-strength of traveling waves for each oscillation cluster. We used a circular-shuffling procedure to test the significance of correlations where we circularly shuffle the wave-strength values with respect to the average amplitude and estimate the correlation on this shuffled data to build a distribution of surrogate correlation values against which the observed correlation was tested (*p* < 0.05) (**Methods**). We found that for ~71% of clusters (20 out of the 28 clusters), there was a statistically significant correlation between amplitude and wave-strength. All, except one, clusters out of these 20 clusters, showed a statistically significant positive amplitude- $\rho_{adj}^{2}$ correlation. Cluster frequency for an oscillation cluster was defined to be the average of the oscillation frequencies across all electrodes for the given oscillation cluster.

| **Subject #** | **Cluster #** | **Cluster frequency** | **Amplitude-**$\rho_{adj}^{2}$  **Pearson *r*** | ***p* < 0.05?**  **(Yes(Y)/No(N))** |
| --- | --- | --- | --- | --- |
| 1 | 1 | 8.08 | 0.182 | Y |
| 1 | 2 | 12.97 | 0.028 | N |
| 1 | 3 | 7.61 | -0.019 | N |
| 1 | 4 | 15.88 | 0.064 | Y |
| 2 | 1 | 8.14 | 0.130 | Y |
| 2 | 2 | 16.69 | 0.154 | Y |
| 2 | 3 | 4.66 | 0.020 | N |
| 3 | 1 | 21.98 | 0.208 | Y |
| 3 | 2 | 6.94 | 0.119 | Y |
| 5 | 1 | 28.37 | 0.097 | Y |
| 5 | 2 | 5.36 | 0.022 | N |
| 6 | 1 | 18.12 | 0.012 | N |
| 6 | 2 | 7.67 | 0.068 | Y |
| 6 | 3 | 17.21 | 0.086 | Y |
| 6 | 4 | 6.68 | 0.181 | Y |
| 7 | 1 | 8.49 | 0.013 | N |
| 7 | 2 | 21.25 | 0.192 | Y |
| 8 | 1 | 6.35 | 0.118 | Y |
| 8 | 2 | 21.00 | 0.260 | Y |
| 8 | 3 | 6.56 | 0.057 | Y |
| 8 | 4 | 22.46 | 0.169 | Y |
| 9 | 1 | 17.46 | 0.203 | Y |
| 9 | 2 | 7.29 | 0.173 | Y |
| 9 | 3 | 16.72 | 0.076 | Y |
| 10 | 1 | 7.79 | -0.080 | Y |
| 10 | 2 | 18.08 | 0.071 | Y |
| 10 | 3 | 7.78 | 0.006 | N |
| 10 | 4 | 16.90 | -0.028 | N |

**Supplementary File 1B.** **Wave-strength (denoted as**$\rho_{adj}^{2}$**) of high (beta) frequency traveling waves is uncorrelated with the wave-strength ($\rho_{adj}^{2}$ (low)- $\rho_{adj}^{2}$ (high)), phase (phase (low)- $\rho_{adj}^{2}$ (high)), and power (power (low)- $\rho_{adj}^{2}$ (high)) of low (theta) frequency traveling waves.** For testing the statistical significance of the observed correlations, values from one time-series were circularly shuffled with respect to the other so that the instantaneous correlation between the two time-series is destroyed, and correlation analysis was repeated on this shuffled data to build a distribution of surrogate correlation values against which the observed correlation was tested (*p* < 0.05) (**Methods**).

| **Subject #** | **Cluster-pair #** | **Cluster-pair frequencies (low (theta), high (beta))** | **$\rho_{adj}^{2}$(low)-$\rho_{adj}^{2}$(high)**  **Pearson *r*** | ***p* < 0.05?**  **(Y/N)** | **Phase (low)-**  **$\rho_{adj}^{2}$(high)**  **Pearson *r*** | ***p* < 0.05?**  **(Y/N)** | **Power (low)-$\rho_{adj}^{2}$(high)**  **Pearson *r*** | ***p* < 0.05?**  **(Y/N)** |
| --- | --- | --- | --- | --- | --- | --- | --- | --- |
| 1 | 1, 4 | 8.08, 15.88 | 0.009 | N | 0.012 | N | 0.00001 | N |
| 1 | 3, 2 | 7.61, 12.97 | -0.025 | N | 0.034 | N | -0.00017 | N |
| 2 | 1, 2 | 8.14, 16.69 | 0.047 | Y | 0.024 | N | -0.00014 | N |
| 3 | 2, 1 | 6.94, 21.98 | 0.023 | N | 0.045 | N | -0.00014 | N |
| 5 | 2, 1 | 5.36, 28.37 | -0.016 | N | 0.054 | N | -0.00018 | N |
| 6 | 2, 1 | 7.67, 18.12 | -0.011 | N | 0.061 | N | -0.00031 | N |
| 6 | 4, 3 | 6.68, 17.21 | 0.002 | N | 0.051 | N | -0.00016 | N |
| 7 | 1, 2 | 8.49, 21.25 | -0.016 | N | 0.040 | N | 0.00010 | N |
| 8 | 1, 2 | 6.35, 21.00 | 0.017 | N | 0.028 | N | -0.00001 | N |
| 8 | 3, 4 | 6.56, 22.46 | -0.009 | N | 0.035 | N | 0.00012 | N |
| 9 | 2, 3 | 7.29, 16.72 | 0.036 | Y | 0.030 | N | -0.00015 | N |
| 10 | 1, 2 | 7.79, 18.08 | 0.001 | N | 0.011 | N | -0.00002 | N |
| 10 | 3, 4 | 7.78, 16.90 | 0.007 | N | 0.016 | N | 0.00010 | N |
